# Infectivity of Symptomatic Patients and Their Contribution for Infectiousness of Mosquitoes following a Membrane Feeding Assay in Ethiopia

**DOI:** 10.1128/spectrum.00628-22

**Published:** 2022-09-06

**Authors:** Andargie Abate, Soriya Kedir, Mitiku Bose, Jifar Hassen, Laurent Dembele, Lemu Golassa

**Affiliations:** a Aklilu Lemma Institute of Pathobiology, Addis Ababa University, Addis Ababa, Ethiopia; b College of Medicine and Health Sciences, Bahir Dar University, Bahir Dar, Ethiopia; c Adama Regional Laboratory, Oromia Region Health Bureau, Adama, Ethiopia; d Adama Science and Technology University, Adama, Ethiopia; e Université des Sciences, des Techniques et des Technologies de Bamako (USTTB), Malaria Research and Training Center (MRTC), Bamako, Mali; University of Virginia

**Keywords:** membrane feeding assay, oocysts, sporozoites, Ethiopia

## Abstract

The membrane feeding assay is widely used to evaluate the efficacy of transmission-blocking interventions (TBIs) and identify the reservoir of malaria. This study aimed to determine the infectivity of blood meals from symptomatic Plasmodium-infected patients to an Anopheles arabiensis colony in Ethiopia. A membrane feeding assay was conducted on a total of 63 Plasmodium falciparum- and/or Plasmodium vivax-infected clinical patients in East Shoa Zone, Ethiopia. Detection of P. falciparum and P. vivax in blood samples was done using microscopy. Mosquito infection rates were determined by dissection of mosquitoes’ midguts, while mosquito infectiousness was observed by dissection of their salivary glands. The proportion of infectious symptomatic patients was 68.3% (43/63). Using the chi-square or Fisher’s exact test, the oocyst infection levels were higher among patients infected with P. vivax, females, and rural residents. Nearly 57% (56.7%, 17/30) of assays produced sporozoites in the salivary glands of mosquitoes. Both oocyst and sporozoite infection rates had positive correlations with parasitemia and gametocytemia. High infectiousness of symptomatic patients was observed, with a greater proportion of infectious mosquitoes per assay. Demonstrating oocyst infection in the mosquitoes might confirm estimates of the infectiousness of mosquitoes, although some of the oocyst-infected mosquitoes failed to produce sporozoites.

**IMPORTANCE** Malaria remains one of the most devastating infectious diseases globally, and transmission-blocking activities are needed. Plasmodium transmission from human to mosquitoes is poorly studied, particularly in endemic countries, and the membrane feeding assay allows it to be determined. In this study, we demonstrated human infectious reservoirs of malaria. Moreover, the effect of Plasmodium-infected patients on the infectiousness of mosquitoes was also observed. These findings are therefore important for designing future evaluation of transmission-blocking interventions that will support the malaria elimination program.

## INTRODUCTION

Malaria remains a challenge to both public health and socioeconomic development globally (1, 2). It is estimated that the human population experienced 241 million cases and 627,000 deaths in 2020 alone (3), indicating that malaria is fighting back against interventions (4). Malaria control is threatened by the spread of insecticide-resistant mosquitoes and drug-resistant parasites (5), and such conditions complicate control measures and, thus, require new innovations blocking the disease transmission.

Efforts to reduce the burden of malaria would be improved by better understanding of malaria transmission dynamics (4). The Plasmodium species are transmitted from human to human by the bite of female Anopheles mosquitoes (6). Malaria transmission from human to mosquito occurs when mosquitoes ingest mature gametocytes circulating in the peripheral blood. The parasite undergoes sexual reproduction and invades the midgut to form oocysts that will be ruptured to release sporozoites that then migrate to mosquito salivary glands. Then, mosquito-to-human transmission occurs when sporozoites are injected into the skin (6–9).

Approaches to reduce the transmission by eliminating the gametocyte reservoir in humans or interfering with sporogonic development of the parasites in mosquitoes are the current focus of control strategies (10). The efficacy of these transmission-blocking interventions (TBIs) (10, 11) can be evaluated by using the membrane-feeding assay (MFA). The MFA is widely accepted for measuring the transmission of malaria (12) by detecting either oocysts in the midgut or sporozoites in the salivary glands of the blood-fed mosquitoes (13). The proportion of mosquitoes infected with oocysts is commonly used as an outcome measure of TBIs (14, 15) due to a positive correlation observed between oocyst and sporozoite infections (16).

Currently, however, taking the intensity of oocysts as the indicator is under question due to a report that high oocyst intensity negatively affected oocyst growth and the number of sporozoites reaching the salivary glands and increased mosquito mortality (17). Thus, oocyst numbers were not correlated with sporozoite loads, particularly when the oocyst load was too high (16), providing contradictory issues for taking oocyst loads as the outcome of TBIs.

The correlation between oocyst and sporozoite intensity is not conclusive; however, a better understanding of the dynamics of the sporozoites’ transition from oocysts can provide insights as to whether detection of oocysts is enough to measure the transmission of malaria, particularly in endemic areas. In addition, knowing the correlation guides the management of malaria, due to the fact that TBIs might be needed to prevent oocyst burdens if oocyst and sporozoite loads have a positive correlation. However, TBIs might be needed to completely eliminate mosquito infection if the oocyst loads have no correlation or if low oocyst loads can result in onward transmission.

Thus, this study assessed the infectivity of symptomatic Plasmodium-infected patients to mosquitoes in Anopheles arabiensis colonies and evaluated the correlations of oocyst and sporozoite infection rates/densities and infectiousness of mosquitoes in East Shoa Zone, Oromia Region, Ethiopia.

## RESULTS

### Characteristics of the study participants.

**FIG 1:**
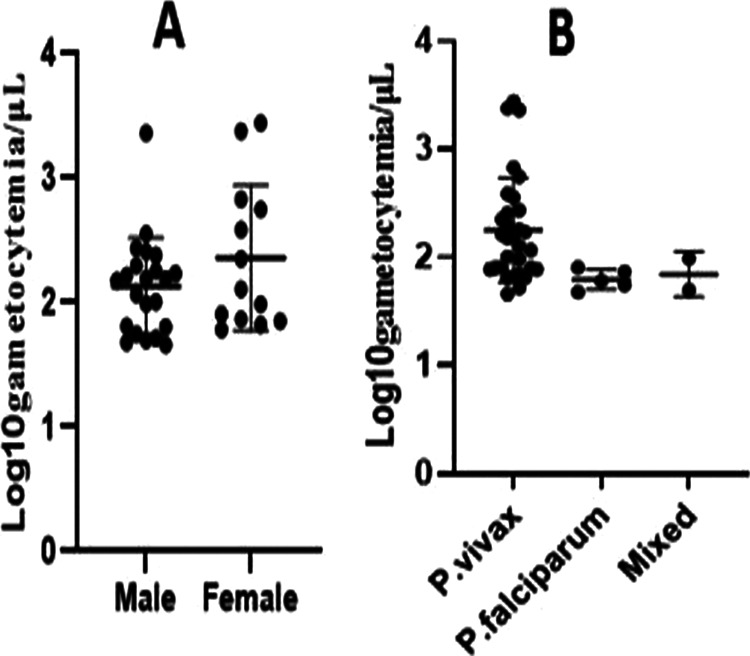
Gametocyte densities (mean values ± standard deviations) related to sex of participants and *Plasmodium* species.

Out of the total of 67 Plasmodium-infected patients recruited, 3 were not included due to problems in the preparation of assays. In addition, dissection was not performed on mosquitoes from one patient’s assay due to a COVID-19 lockdown. Thus, the feeding assays were done successfully for 63 patients, of whom 71.4% were males and 35 (55.6%) were ever married (married, divorced, and separated). The majority (66.7%) of the participants came from Adama City Administration (seven health centers and one malaria diagnostic center), while the remaining 33.3% were from out of Adama City (five health centers). The largest proportion (71.4%) of study participants were infected with Plasmodium vivax. The median values (ranges) were 4,098 (111 to 19,582) parasites/μL for parasite density and 71 (0 to 2,785) gametocytes/μL for gametocytemia (Table 1). The gametocyte densities were higher among P. vivax-infected and female patients (Fig. 1). The mean values (ranges) of gametocyte densities among urban and rural residents were 160 (46 to 2,395) and 199 (48 to 2,785), respectively.

**TABLE 1:** Demographic characteristics and malaria diagnosis of study participants through microscopy in East Shewa Zone, Ethiopia

Variable	Category	Frequency (%) or median (range)
Sex	Male	45 (71.4)
	Female	18 (28.6)

Age	<24	22 (34.9)
	25–44	25 (39.7)
	>45	16 (25.4)

Marital status	Never married	28 (44.4)
	Ever married	35 (55.6)

Residence	Adama City	42 (66.7)
	Out of Adama City	21 (33.3)

Plasmodium species	P. vivax	45 (71.4)
	P. falciparum	15 (23.8)
	Mixed P. vivax and P. falciparum	3 (4.8)

No. of asexual parasites/μL		4,098 (111–19,582)
Gametocyte density/μL		71 (0–2,785)

### Characteristics of mosquitoes and their feeding success rates.

For every membrane feeding assay, an average of 175 (ranging from 40 up to 550) female adult mosquitoes were exposed to Plasmodium-infected blood. Overall, the mosquito feeding rate was 61.8% (6,795/11,000, ranging from 18 to 330 mosquitoes per assay) of mosquitoes. In relation to Plasmodium species, 62.1% (5,230/8,420), 64.0% (1,369/2,140), and 44.5% (196/440) of mosquitoes were engorged after their exposure to blood samples from P. vivax-, P. falciparum-, and mixed-species-infected patients, respectively (Table 2).

**TABLE 2:** Summary details of membrane feeding assay in East Shewa Zone, Ethiopia

Characteristic	Value for:			
	P. vivax	P. falciparum	Mixed Plasmodium species	Total
No. of:				
Exposed mosquitoes	8,420	2,140	440	11,000
Fed mosquitoes	5,230	1,369	196	6,795

Age (days) range of exposed mosquitoes (mean ± SD)	2–8 (4.49 ± 1.40)			NA^*a*^
Starvation period (h) ± SD (range)	11:45 ± 3.85 (4–20)			NA

% (no. with characteristic/no. tested) of:				
Infectious individuals	77.8 (35/45)	46.7 (7/15)	33.3 (1/3)	68.3 (43/63)
Infected mosquitoes	30.3 (271/895)	6.7 (29/431)	2.8 (2/72)	21.6 (302/1,398)

a NA, not applicable.

### Human-to-mosquito transmission.

An average of 23 (range, 5 to 60) mosquitoes per assay was dissected 7 days postinfection. More than half (68.3%, 43/63) of the participants were infectious to at least one mosquito, while the remaining 31.7% (20/63) failed to produce oocyst infections. Among P. vivax-infected participants, a higher (77.8%) proportion of blood samples were infectious to mosquitoes, whereas only 46.7% of blood samples from P. falciparum infected participants gave rise to mosquito infections (Table 2). Interestingly, females, rural residents, and P. vivax-infected participants were more infectious to mosquitoes than males, urban residents, and P. falciparum-infected participants, respectively (Table 3).

**TABLE 3:** Presence of oocyst-positive mosquitoes by characteristics of participants in East Shewa Zone, Ethiopia

Variable	Category	No. (%) having at least one mosquito with oocyst(s)		χ^2^
		No	Yes	
Age	≤24	8 (36.4)	14 (63.6)	0.057
	25–44	4 (16.0)	21 (84.0)	
	≥45	8 (50.0)	8 (50.0)	

Sex	Male	18 (40.0)	27 (60.0)	0.036^*a*^
	Female	2 (11.1)	16 (88.9)	

Marital status	Never married	11 (39.3)	17 (60.7)	0.250
	Ever married	9 (25.7)	26 (74.3)	

Residence	Urban	17 (40.5)	25 (59.5)	0.046^*a*^
	Rural	3 (14.3)	18 (85.7)	0.03^*a*^

Plasmodium spp.	P. vivax	10 (22.2)	35 (77.8)	
	P. falciparum	8 (53.3)	7 (46.7)	
	Mixed species	2 (66.7)	1 (33.3)	

a Fisher’s exact test.

### Midgut infection rate.

**FIG 2:**
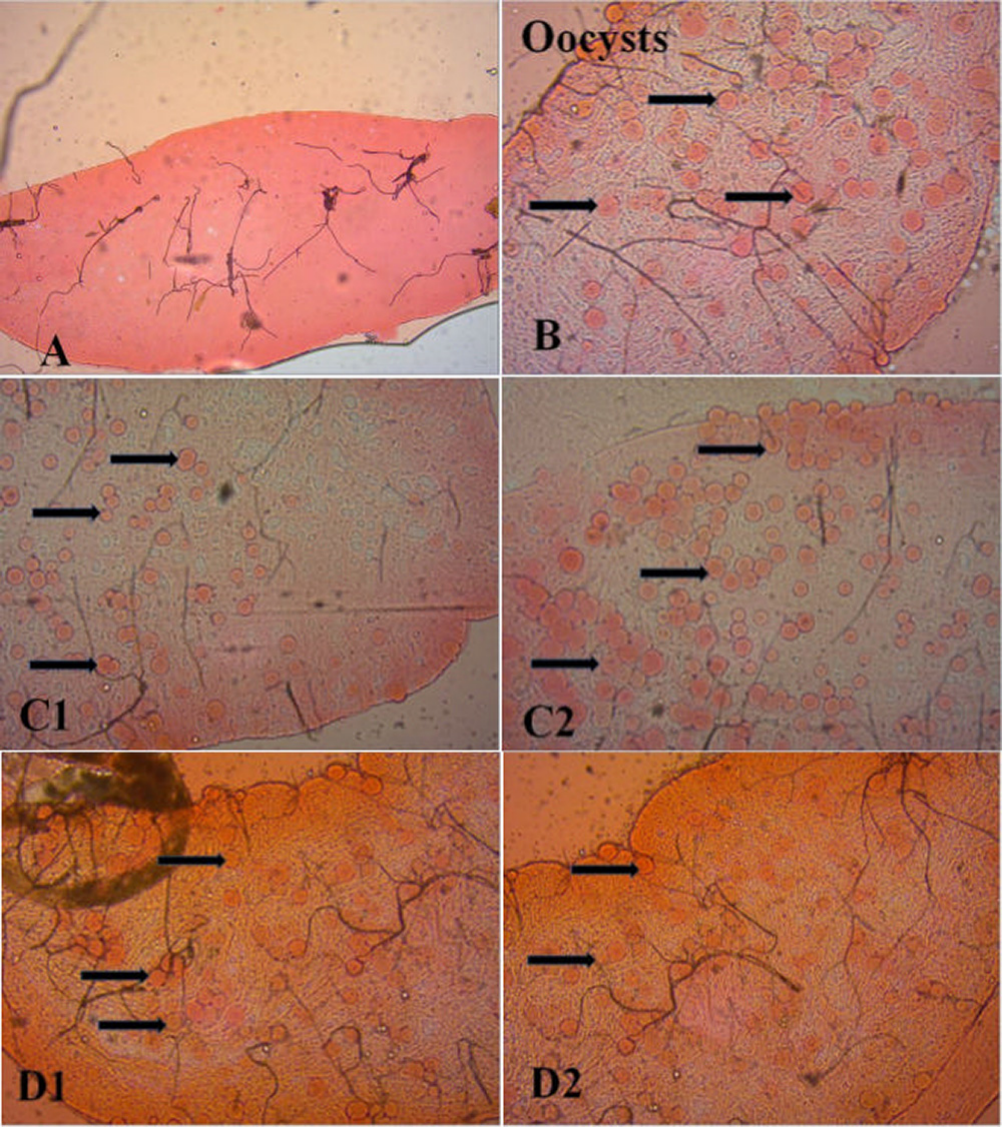
Images of midguts of dissected P. vivax-infected blood-fed mosquitoes. Shown are midguts from one mosquito negative for oocysts (A), one mosquito positive for oocysts (B), one mosquito positive for oocysts from one patient (C1 and 2), and one mosquito positive for oocysts from another patient (D1 and 2).

**FIG 3:**
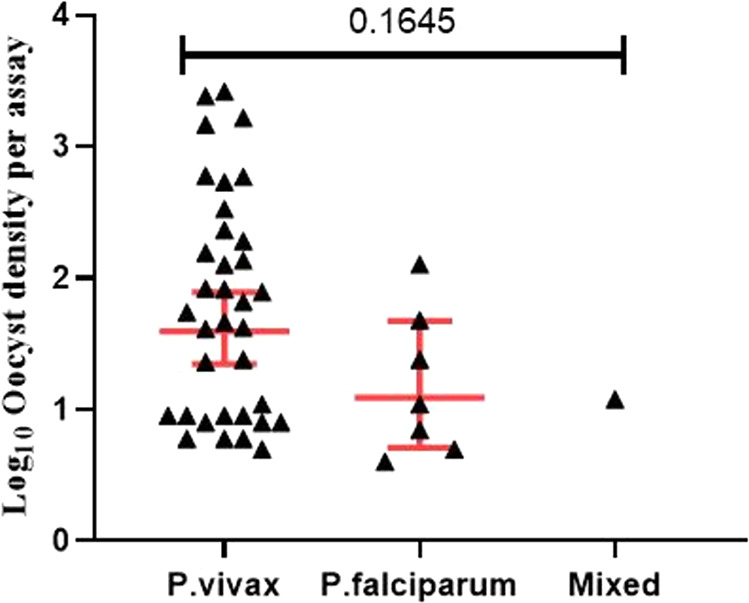
Oocyst densities (mean values [Log10 transformed] ± standard deviations) of midguts per infectious individual based on *Plasmodium* species.

Figure 2 shows an example of a mosquito midgut infected with P. vivax oocysts. Out of all the dissected mosquitoes, 21.6% (302/1,398) were infected with microscopically detected oocysts (Table 2), with a geometric mean of nearly 8 oocysts per mosquito (ranging from 1 to 285 oocysts/midgut). Moreover, the median oocyst load per assay was 42, ranging from 4 up to 2,625 per participant. The oocyst loads were relatively higher among feeding assays done using blood from P. vivax-infected patients (Fig. 3), and the highest number of 2,625 oocysts per experiment was observed among P. vivax-infected patients.

### Salivary gland infection.

**FIG 4:**
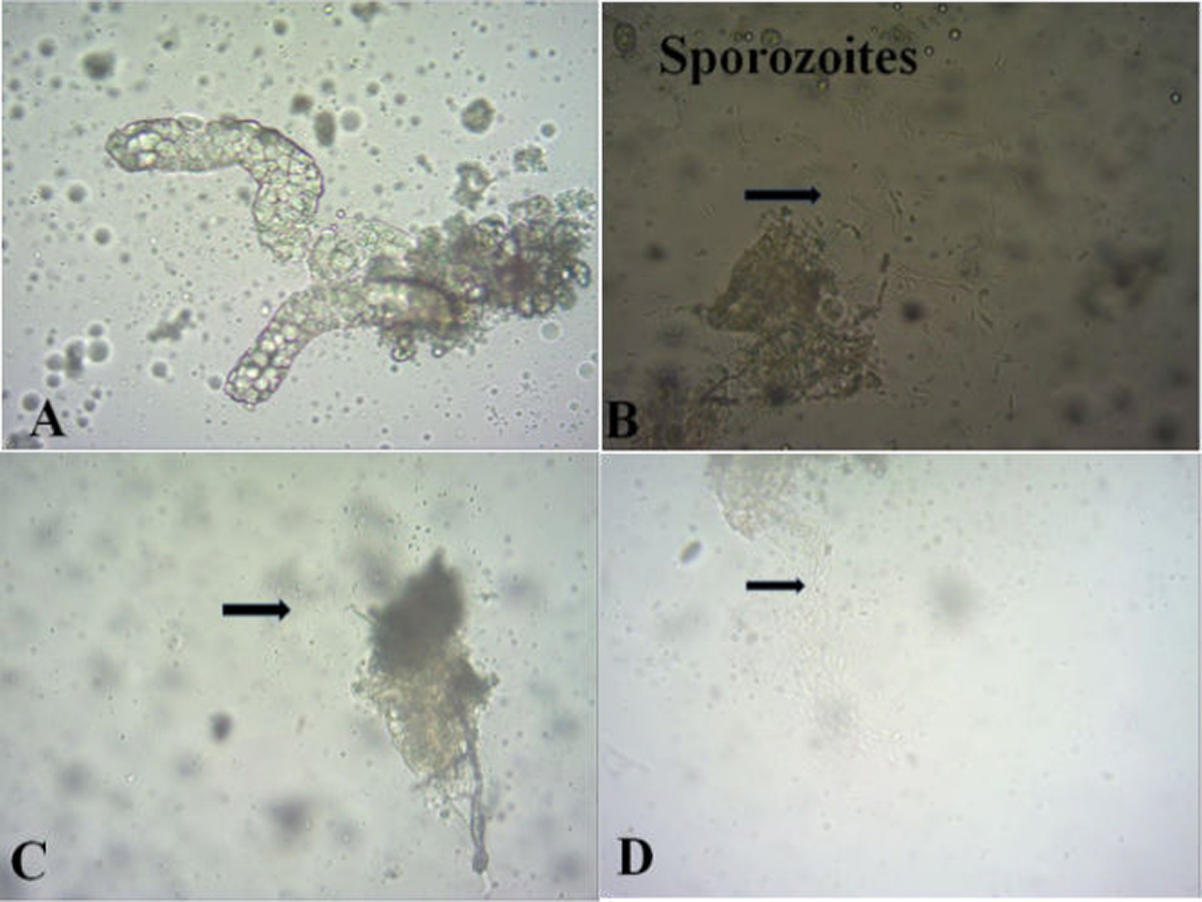
Examples of images of salivary glands dissected from mosquitoes and sporozoite densities per dissected mosquito from P. vivax-infected patients. Sporozoite-negative mosquito for patient 1 (A), sporozoite-positive mosquito for patient 2 (B), sporozoite-positive mosquito for patient 3 (C), and sporozoite-positive mosquito for patient 4 (D).

Salivary gland dissection was performed on mosquitoes fed on blood samples from 28 randomly selected participants among the 43 infectious participants, as well as on mosquitoes fed on blood samples from 2 (10%) noninfectious participants. More than half (56.7%, 17/30) of the participants were infectious for at least one mosquito, with sporozoites detected. Salivary gland infection was not observed from all P. falciparum-infected participants or from participants with oocyst-negative mosquitoes. Out of the assays with oocyst-positive mosquitoes, 60.7% (17/28) had sporozoite-positive mosquitoes. Due to the problem of survival of mosquitoes until salivary gland dissection time, only 284 mosquitoes were dissected. Figure 4 shows an example of a mosquito salivary gland infected with P. vivax sporozoites. The salivary gland infection rate was 20.4% (58/284 of dissected mosquitoes), whereas the remaining 79.6% (226/284) failed to produce sporozoite infection. The sporozoite loads ranged from 56 to 1,337, with a mean of 320 sporozoites per assay. The numbers of sporozoites ranged from 56 to 1,253, with a median of 92 sporozoites per salivary gland.

### Relationship of infections and densities of oocysts and sporozoites.

**FIG 5:**
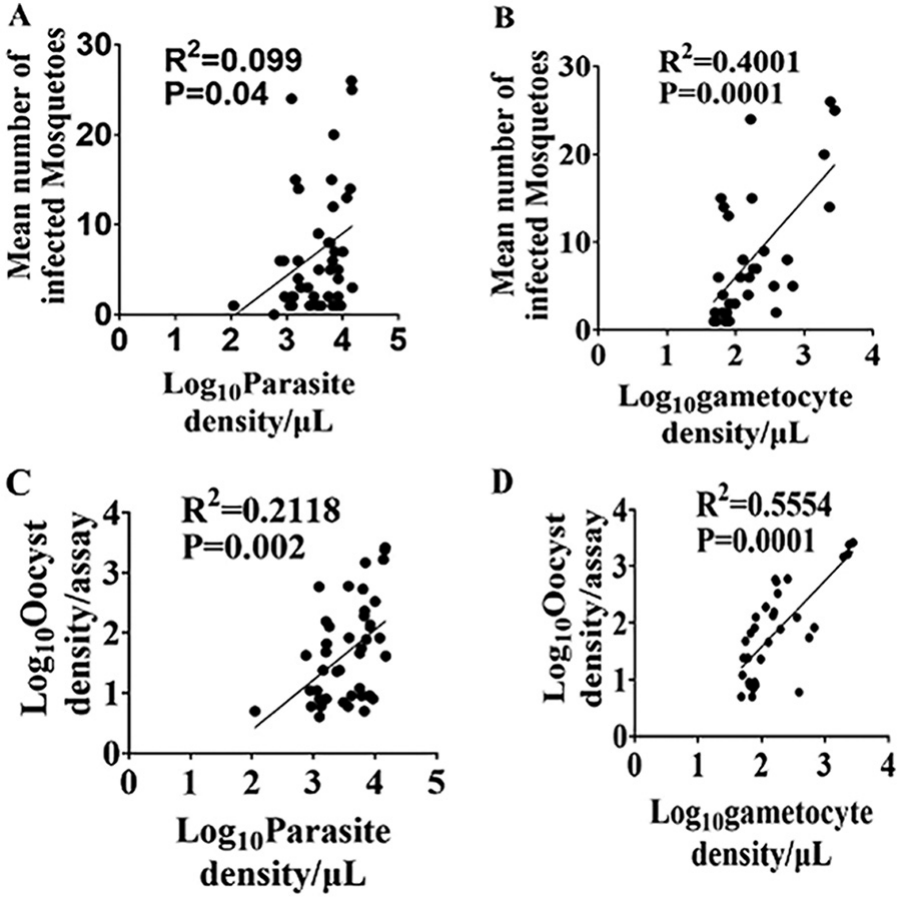
Relationships between the oocyst infection and mean oocyst density of each membrane feeding assay and *Plasmodium* parasite and gametocyte densities in blood.

The oocyst infection rates of mosquitoes had statistically significant correlations with parasitemia and gametocytemia (*R*^2^ = 0.099, *P* = 0.04 and *R*^2^ = 0.41, *P* = 0.0001, respectively). The oocyst loads were also significantly correlated with parasitemia (*R*^2^ = 0.3322, *P* = 0.0001) and gametocytemia (*R*^2^ = 0.8999, *P* = 0.0001) (Fig. 5).

**FIG 6:**
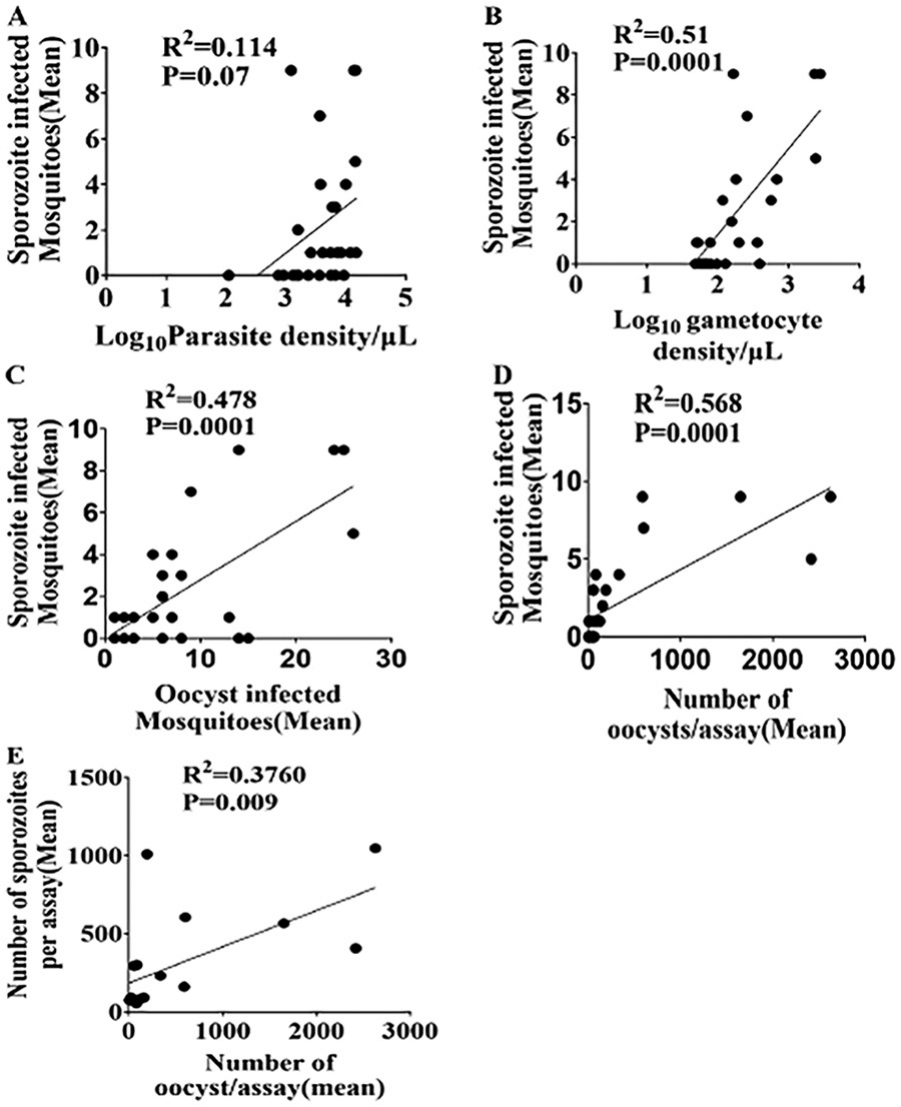
Relationships between the sporozoite infection and mean sporozoite density of each membrane feeding assay and *Plasmodium* parasite and gametocyte densities in blood, oocyst infection, and mean density.

Statistically significant correlations were observed between the mean numbers of mosquitoes infected with sporozoites and gametocytemia (*R*^2^ = 0.51, *P* = 0.0001), oocyst infection rates (*R*^2^ = 0.478, *P* = 0.0001), and oocyst loads (*R*^2^ = 0.568, *P* = 0.0001). When oocyst and sporozoite loads were compared, a positive correlation (*R*^2^ = 0.376, *P* = 0.01) was observed. The sporozoite loads were also positively correlated with gametocyte levels (*R*^2^ = 0.341, *P* = 0.01). However, the parasite densities were not significantly correlated with sporozoite infections (*R*^2^ = 0.114, *P* = 0.07) and sporozoite loads (*R*^2^ = 0.087, *P* = 0.25) (Fig. 6). The numbers of mosquitoes that had their salivary glands dissected had no significant correlation with sporozoite infection rates (*P* = 0.07) (data not shown).

## DISCUSSION

The membrane-feeding method has been widely used to measure the potential transmission of Plasmodium parasites from humans to mosquitoes (18, 19) and mosquitoes to humans (16). It avoids the difficulty of recruiting volunteers for direct mosquito feeding (20, 21). Estimating the infectiousness of the Plasmodium reservoir to mosquitoes and mosquitoes’ infectiousness to humans is crucial to understand the epidemiology of malaria and its changes after the application of control measures (22, 23).

The current study, thus, has been done to detect oocysts and sporozoites and their intensities in midguts and salivary glands, respectively, dissected from mosquitoes. The study successfully demonstrated that the proportion of infectious symptomatic patients was 68.3%. The current finding is relatively higher than those of some previous studies (32.6% to 57.1%) (22, 24, 25) but lower than the results (85.7% to 100%) elsewhere (18, 26). These differences might be due to variations in immune response, gametocyte density, and susceptibility of vector species to infection (27).

This study observed that the proportion of infectious patients infected with P. vivax was higher (77.8%) than the proportion of infectious patients infected with P. falciparum (46.7%). This finding is in line with previous studies reporting that P. vivax patients were more infectious than P. falciparum-infected patients (34.9% versus 15.1% and 42% versus 9.2%, respectively) (28, 29). This could be due to the higher gametocyte density in P. vivax- than in P. falciparum-infected patients in the current study. Furthermore, it might be due to the biology of the parasites, where the sexual stages of P. vivax appear in the blood earlier than those of P. falciparum (30). In addition, P. vivax gametocytes are transmitted more efficiently and are transmissible at lower parasite densities than those of P. falciparum (31).

We observed that females and rural residents represented the major reservoirs of Plasmodium parasites. This was because infection rates of mosquitoes among females and rural residents in this study were significantly higher than in the corresponding males and urban residents, respectively. It might be because the gametocyte densities were higher among females and rural residents in the current study. This is also supported by a previous study done by Coalson et al., who reported that the gametocyte carriage rates and densities were higher among those living in unfinished houses (32). In addition, most of the females in the current study were infected with P. vivax.

The mosquito infection rate was higher (21.6%) in the current study than previous findings elsewhere (1.8% to 19.0%) (24, 28, 33). We found that parasitemia and gametocytemia were good predictors of infectivity. That is, the higher the numbers of asexual and sexual parasites, the more mosquitoes were infected during the MFA. Our findings are in line with previous studies where a positive correlation was observed between the mosquito infection rates and the parasite densities (29, 34, 35) but contradictory to a study done by Balabaskaran Nina et al., who demonstrated the absence of correlation between the oocyst infection rates and parasitemia and gametocytemia (16).

High oocyst densities per dissected mosquito and also per membrane feeding assay were recorded in the current study, particularly from P. vivax-infected patients. This might be due to higher gametocyte densities in P. vivax-infected participants. It could be due to the observed fact that the mean oocyst density increased with blood parasitemia and gametocytemia, in agreement with previous studies (7, 16, 36, 37).

A previous study (35) and this study have shown that the detection of oocysts in the mosquito’s midgut is a predictor of mosquito infectivity, although some oocysts fail to release sporozoites. The current study found that 60.7% of the assays with oocyst-positive mosquitoes had sporozoite-positive mosquitoes. Although the number of mosquitoes whose salivary glands were dissected was small, this highlights the fact that more than half of the assays of symptomatic patients resulted in infectiousness of mosquitoes. This is because the presence of sporozoites in salivary glands of mosquitoes had a great contribution to onward mosquito-to-human transmission (8). The infectiousness of mosquitoes varied based on the Plasmodium species, as shown by the fact that a higher proportion (94.1%) of salivary gland infection was detected from assays done using blood samples from P. vivax-infected patients. This finding is in line with the observations of Moreno and colleagues, who reported that 95% of salivary gland-dissected mosquitoes had sporozoites (38). However, it was higher than a previous study elsewhere that documented 48.5% of mosquitoes as salivary gland infected (39).

It has been demonstrated previously that the percentages of oocyst infection were significantly correlated with the percentages of sporozoite infection (15, 16, 39), which is in agreement with the current study. These findings might also provide significant support for the development and evaluation of drugs and/or vaccines (38) challenging liver stages of the parasite, because laboratories conducting vaccine and/or drug studies are all located in nonendemic countries, and thus, this supports the need to get sporozoite stages of the parasites from endemic settings. Thus, the demonstration of oocyst infection in the mosquitoes might predict the estimates of infectiousness of mosquitoes without the need for salivary gland dissection, which requires an extra week for the assay.

In the study presented here, there was a strong correlation between sporozoite infection and gametocytemia, which is in contrast with a previous study in India (16). The significant correlation between oocyst numbers and sporozoite loads found is in agreement with previous studies (16, 40, 41), but a negative correlation has also been reported when the oocyst load was greater than 79 per individual mosquito (16).

The limitations of this study include the failure to use molecular laboratory techniques to determine parasite and gametocyte densities. The immunological, genetic, nutritional, and health status (e.g., occurrence of coinfections) of the study participants, which could affect the parasite levels, were not considered. In addition, the number of malaria cases was too small for making firm conclusions regarding predictors of mosquito infections.

### Conclusion.

Overall, the current study reported high infectiousness of symptomatic patients, and the infectiousness was affected by the gametocyte and parasite densities. The findings might be an indicator to focus on the reservoirs when considering control and elimination strategies. Further studies on the human- and mosquito-related determinants of mosquitoes’ infection using large sample sizes and molecular techniques and minimum thresholds for oocyst and sporozoite loads for onward transmission are thus needed.

## MATERIALS AND METHODS

### Study area and period.

The study was conducted in Adama City and its surroundings, located 100 kilometers southeast of Addis Ababa, the capital city of Ethiopia, from October 2019 to January 2021. Adama lies 1,623 m above sea level and has an annual temperature of 20.5°C, and about 808 mm of precipitation falls annually. Adama City and its surrounding area have weather conditions conducive for malaria transmission, and both P. vivax and P. falciparum were reported in the area (28, 42). For this study, patients were recruited from 12 health centers (7 in the Adama City Administration and 5 from surrounding areas) and the Adama malaria diagnostic center. All health institutions were within a 25-km radius of the Adama City Administration.

### Study population.

The study population included patients aged ≥5 years with self-reported history of fever visiting the health institutions. In this study, all patients known to have uncomplicated malaria, confirmed by microscopy, during the study period were included. However, pregnant women and patients with a recent history of antimalarial drugs were excluded from the study.

### Ethics approval and consent to participate.

The study was conducted in accordance with the Declaration of Helsinki and approved by the Institutional Review Board of Addis Ababa University, Aklilu Lemma Institute of Pathobiology (reference no. ALIPB IRB/18/2012/20), and the National Research Ethics Review Committee (reference no. MoSHE/02/152/778/21). Informed consent was obtained from all participants. The study participants were informed that they could be treated according to the national treatment guidelines if positive for any Plasmodium parasite. The participants and/or their guardians were given the right to refuse to take part in the study, as well as to withdraw at any time during the study. No names or identifying information were indicated on the questionnaires, and all subjects were assured of confidentiality throughout the study.

### Detection of *Plasmodium* infection.

Finger prick blood samples were collected after cleaning the finger surface with alcohol-soaked sterile cotton. Both thin and thick blood films were prepared in a single slide from fresh blood samples. The thin smear was fixed by methanol, and both thin and thick blood films were stained with 2% Giemsa solution. Thick blood smears were used to detect the presence of parasites, while thin blood films were used to determine the species of the parasites. Finally, the dried blood films were examined under a microscope with a 100× oil immersion lens objective. Slides were double read by two different experienced microscopists blindly. Slides with discordant results were read by a third reader blindly, and the majority results were taken as final. The parasite density was determined by considering a total of 8,000 white blood cells (WBC)/μL using the following formula: (number of parasites counted × 8,000)/number of WBC counted. Then, the parasitemia was determined by averaging the results of the two independent readers.

### Sample collection and processing.

After the patients were confirmed positive for malaria by microscopy at each health institution, they were recruited and transported to the mosquito infection laboratory at the Adama public health research and referral laboratory insectary center. After the arrival of the patients at the mosquito infection laboratory, approximately 5 mL of venous blood was collected from each study participant in heparinized tubes. The collected blood samples were immediately allowed for mosquito feeding irrespective of gametocyte screening to include all potentially infectious individuals, because of a limitation of microscopy to detect gametocytes.

### Mosquito MFA.

Mosquito membrane feeding assays were conducted using colonies of Anopheles arabiensis that were reared in the Adama public health research and referral laboratory insectary center. The insectary was maintained at 24 to 27°C and a relative humidity of 70 to 90%. Starved female Anopheles mosquitoes aged 2 to 8 days were collected and distributed into paper cups (40 mosquitoes per cup) that were covered with a mosquito net. The glass membrane feeder was immediately filled with fresh blood, and then the mosquitoes were exposed to blood and allowed to feed through a Parafilm for 30 min to 1 h in a dark place. During blood feeding, a constant 37°C circulating water system was maintained to prevent exflagellation of the microgametocytes. One hour after the feeding experiment, female mosquitoes that were not engorged with a blood meal were removed, while the blood-fed mosquitoes were transferred to the big mosquito-rearing cages and maintained under optimum biosafety standards under the temperature and humidity conditions described above. Cotton wool impregnated with sucrose solution was put on each cage and replaced daily until the dissection time, as described previously (14).

### Mosquitoes’ infection and infectiousness.

Seven to 8 days postinfection, the mosquitoes were immobilized by placing them in a −20°C freezer for 10 min. Then, dissection of mosquitoes’ midguts was performed to determine the infection rates by using a drop of 1% mercurochrome on a slide. The midgut and mercurochrome were covered with a coverslip. Finally, each mosquito’s midgut was examined microscopically for the presence of oocysts at ×20 magnification. Samples (25% of the mosquitoes available during dissection time) of randomly selected blood-fed mosquitoes from each assay were dissected to observe the oocyst infection. Once oocysts were detected, the number of oocysts was counted and recorded for each individual mosquito. The remaining 75% of the mosquitoes were maintained for an additional 7 or 8 days (14 or 15 days post-blood feed) for salivary gland dissection. The salivary gland dissection was conducted using a drop of phosphate-buffered saline (PBS) (1× PBS) following the standard protocol (14). The number of sporozoites was counted and recorded for each individual mosquito by using a microscope.

### Data management and analysis.

The data were entered into EpiData version 3.1 and exported to Statistical Package for Social Sciences (SPSS) version 25 for analysis. All the graphs were produced using GraphPad Prism software version 8.0. The chi-square (χ^2^) and/or Fisher’s exact test was applied to assess associations between proportions of mosquitoes infected and some demographic characteristics of the participants. Linear regression was used to assess the correlations between successful mosquito infections (i.e., oocyst-infected mosquitoes and/or sporozoite-infected mosquitoes) and gametocytemia and parasitemia. The correlation between mean oocyst loads and mean sporozoite loads was also tested using linear regression. A *P* value of <0.05 was considered statistically significant in this study.

### Data availability.

The data that support the findings of this study are available from the corresponding author on reasonable request. All relevant data are within the manuscript.
